# The red fluorescent protein eqFP611: application in subcellular localization studies in higher plants

**DOI:** 10.1186/1471-2229-7-28

**Published:** 2007-06-06

**Authors:** Joachim Forner, Stefan Binder

**Affiliations:** 1 Molekulare Botanik, Universität Ulm, Albert-Einstein-Allee 11, 89069 Ulm, Germany

## Abstract

**Background:**

Intrinsically fluorescent proteins have revolutionized studies in molecular cell biology. The parallel application of these proteins in dual- or multilabeling experiments such as subcellular localization studies requires non-overlapping emission spectra for unambiguous detection of each label. In the red spectral range, almost exclusively DsRed and derivatives thereof are used today. To test the suitability of the red fluorescent protein eqFP611 as an alternative in higher plants, the behavior of this protein was analyzed in terms of expression, subcellular targeting and compatibility with GFP in tobacco.

**Results:**

When expressed transiently in tobacco protoplasts, eqFP611 accumulated over night to levels easily detectable by fluorescence microscopy. The native protein was found in the nucleus and in the cytosol and no detrimental effects on cell viability were observed. When fused to N-terminal mitochondrial and peroxisomal targeting sequences, the red fluorescence was located exclusively in the corresponding organelles in transfected protoplasts. Upon co-expression with GFP in the same cells, fluorescence of both eqFP611 and GFP could be easily distinguished, demonstrating the potential of eqFP611 in dual-labeling experiments with GFP. A series of plasmids was constructed for expression of eqFP611 in plants and for simultaneous expression of this fluorescent protein together with GFP. Transgenic tobacco plants constitutively expressing mitochondrially targeted eqFP611 were generated. The red fluorescence was stably transmitted to the following generations, making these plants a convenient source for protoplasts containing an internal marker for mitochondria.

**Conclusion:**

In plants, eqFP611 is a suitable fluorescent reporter protein. The unmodified protein can be expressed to levels easily detectable by epifluorescence microscopy without adverse affect on the viability of plant cells. Its subcellular localization can be manipulated by N-terminal signal sequences. eqFP611 and GFP are fully compatible in dual-labeling experiments.

## Background

Since the cloning of the green fluorescent protein (GFP) cDNA and its first heterologous expression in the early 1990s [1,2], the use of intrinsically fluorescent proteins (IFPs) has become one of the most powerful tools in molecular and cell biology. These proteins are applied as reporters in gene expression studies, as indicators of intra-cellular physiological changes, for monitoring dynamics of organelles and proteins, for investigation of protein-protein interactions *in vivo *and as fusion partners in studies of the subcellular localization of proteins [3,4].

From the very beginning, many efforts have been made to optimize various features of the native GFP with the aim to improve its application in biological research. These modifications include for instance improved folding efficiency, higher expression level or increased solubility [3]. Cyan and yellow fluorescent derivatives of GFP have been created for investigations requiring the simultaneous distinguishable tagging of more than one protein at a time [5,4]. These are used to compare the spatial distribution or the expression pattern of two or more proteins and for the analysis of protein-protein interactions by FRET. So far no red fluorescent variant of GFP has been reported. Recently, investigation of several non-bioluminescent anthozoan species has led to the isolation of various true red fluorescent proteins (RFPs) [6]. Among these, DsRed and its derivatives are the most commonly used in molecular and cell biological research [7].

Since plants contain a large number of multi-gene families, comparisons of the subcelluar localizations of the individual members are necessary as part of the comprehensive analysis of these proteins. The possibility to label several proteins with different fluorescent proteins is a great advantage when analyzing their respective subcellular localization. As a crucial prerequisite for such studies, the compartments to which the fusion proteins are targeted have to be unequivocally identified. This is often done by staining with compartment-specific dyes. Mitochondria for instance can be visualized by staining with the red fluorescent dye MitoTracker^® ^Red CM-H2Xros (Molecular Probes, Eugene, OR) which specifically interacts with the respiratory chain. The staining procedure, however, is time-consuming, invasive and short-lived and can be replaced simply by co-expression of a spectrally different second fusion protein with a defined subcellular localization. Additionally, the fused target sequence of the fluorescent marker protein can be readily exchanged, which allows selective labeling of nearly every subcellular structure under investigation without the need to have a specific dye for the different compartments.

Despite the discovery of a multiplicity of fluorescent proteins in the red spectral range in recent years [6], so far almost exclusively different forms of DsRed have been used for studies in molecular cell biology in plants [8-12]. These proteins are applied in dual-labeling experiments together with GFP or alone to report on promoter activity or as a marker in transgenic plants. To introduce an alternative RFP for the application in plant cells and to expand the palette of red fluorescent reporters for plant research, we tested the suitability of the red fluorescent protein eqFP611 from the sea anemone *Entacmaea quadricolor *as a marker in subcellular localization experiments in plants.

eqFP611 shows far-red fluorescence with excitation and emission maxima at 559 nm and 611 nm, respectively, and therefore exhibits an extraordinarily large Stokes shift of 52 nm [13]. In contrast, the respective values for DsRed are 558 nm, 583 nm and 25 nm, respectively [13]. Both eqFP611 and DsRed have comparable molecular masses of 25.93 kDa and 26.05 kDa, respectively, for the monomers. The extinction coefficient of eqFP611 (78,000 M^-1^* cm^-1^) is slightly higher than that of DsRed (75,000 M^-1^* cm^-1^). Fluorescence quantum yields for eqFP611 and DsRed are 0.45 and 0.7 and the photobleaching quantum yields are 3.5 * 10^-6 ^and 0.8–9.5 * 10^-6^, respectively. Similar to DsRed, the emission of eqFP611 is constant between pH 4 and 10. Though both form tetramers at physiological concentrations, eqFP611 has a reduced tendency to oligomerize and aggregate as compared to DsRed. With a maturation half-time t_0.5 _of 4.5 h at 24.5°C [14], fluorophore maturation of eqFP611 is much faster than that of DsRed (t_0.5 _> 24 h at 24.5 °C) [13].

We demonstrate that native eqFP611 can be expressed in plant cells. Fusions of this protein with respective N-terminal signal sequences can be efficiently targeted to mitochondria and peroxisomes. We performed co-expression experiments with eqFP611 and GFP and created vectors for the straightforward application of the eqFP611 gene in plants.

## Results and Discussion

### eqFP611 can be functionally expressed in plant cells

Recently, eqFP611, the gene for a red fluorescent protein from the sea anemone *Entacmaea quadricolor*, has been cloned and characterized [13,14]. This protein has been succesfully expressed in bacteria and animal cells [13], but has not yet been tested in plants.

**Figure 1:**
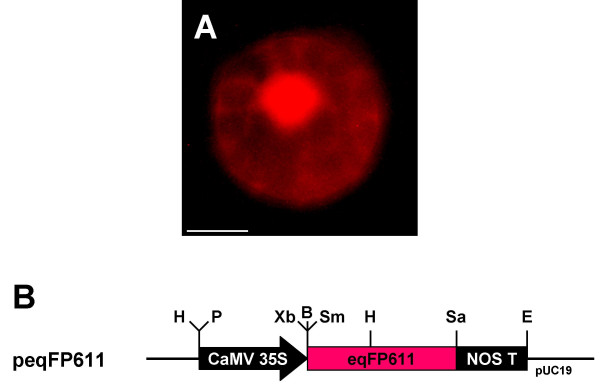
**eqFP611 without presequence**. Transient expression of original eqFP611 without presequence in *N. tabacum *wild-type protoplasts. (A) Image taken through MitoTracker filter set. Scale bar: 10 μm. (B) Plasmid peqFP611 used for transfection. Black arrow, CaMV 35S: cauliflower mosaic virus 35S promoter; red box, eqFP611: eqFP611 coding sequence; black box, NOS T: nopaline synthase terminator. H: HindIII, P: PstI, Xb: XbaI, B: BamHI, Sm: SmaI, Sa: SacI, E: EcoRI restriction sites. Vector backbone: pUC19.

To test its use as a marker in plants, the native eqFP611 cDNA was cloned into a pUC19-based vector. In the resulting plasmid peqFP611, expression of this gene is governed by the strong constitutive cauliflower mosaic virus 35S promoter (CaMV 35S) and the nopaline synthase terminator (NOS T) sequences. Upon inspection of *Nicotiana tabacum *mesophyll cells transfected with this plasmid in the epifluorescence microscope, the red fluorescence was clearly detectable with a filter set (HQ545/30/HQ 610/75) usually used for visualization of MitoTracker Red and here later referred to as MitoTracker filter set (Fig. 1). The protein accumulates in the nucleus and in the cytosol, where it is evenly distributed and does not form any visible aggregates, but is clearly absent from the chloroplasts. No such fluorescence was detectable in untransfected control cells, confirming that the red fluorescence indeed originates from the expression of the introduced eqFP611. Protoplasts were analysed 16 hours after transfection. Incubation for an additional 24 hours did not markedly increase the intensity of the red fluorescence, suggesting the maximal level of mature protein to be essentially reached within 16 hours after transfection. Protoplasts expressing eqFP611 looked perfectly normal and did not show any detrimental effects of this fluorescent protein.

These results show that eqFP611 can be readily used in plants, since the functional protein accumulates to detectable levels without any obvious adverse effects. In contrast to GFP, whose original jellyfish-derived cDNA was misspliced specifically in plants at a cryptic splice site [15], no modification of the eqFP611 coding sequence is necessary for efficient expression in plants.

As expected from its spectral characteristics, the fluorescence is easily detectable with a filter set (see above) that excludes the red autofluorescence of chlorophyll, a crucial advantage for an RFP applied in mesophyll cells. Similar to GFP [16], the native eqFP611 accumulates in the nucleus and in the cytosol in plant cells. Thus, it should be suited to investigate protein targeting into e.g. mitochondria, peroxisomes and plastids within plants. In HeLa cells, native, unmodified eqFP611 was also found in the nucleus and the cytosol [13].

### Targeting eqFP611 to mitochondria

**Figure 2:**
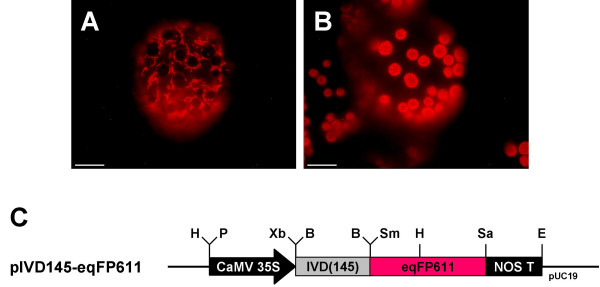
**Mitochondrially targeted eqFP611**. *N. tabacum *wild-type protoplast expressing a fusion protein of eqFP611 and the N-terminal 48 amino acids of IVD. Pictures showing the same cell were taken through MitoTracker (A) and FITC (B) filter sets, respectively. Scale bars: 10 μm. (C) Map of plasmid pIVD145-eqFP611 used for transfection. Black arrow, CaMV 35S: cauliflower mosaic virus 35S promoter; grey box, IVD(145): N-terminal 145 nucleotides of the IVD coding sequence; red box, eqFP611: eqFP611 coding sequence; black box, NOS T: nopaline synthase terminator. H: HindIII, P: PstI, Xb: XbaI, B: BamHI, Sm: SmaI, Sa: SacI, E: EcoRI restriction sites. Vector backbone: pUC19.

To investigate whether eqFP611 can indeed be used as reporter protein for the analysis of subcellular protein sorting, import into plant mitochondria was exemplarily tested. To this end, the presequence of the mitochondrial isovaleryl-CoA-dehydrogenase (IVD) was added to the N-terminus of eqFP611 (plasmid pIVD145-eqFP611). The IVD presequence was chosen because it has previously been found to efficiently target a GFP fusion protein exclusively to mitochondria [17]. In addition, the protein has been repeatedly detected in proteomic analyses of this organelle, demonstrating its unambiguous localization in mitochondria [18-20]. Inspection of the protoplasts transfected with pIVD145-eqFP611 using the MitoTracker filter set revealed the red fluorescence to be restricted exclusively to rod-shaped structures of 1 – 2 μm in length distributed throughout the cell (Fig. 2A). This pattern is characteristic for a mitochondrial localization of the fusion protein. No red fluorescence was detectable in other parts of the protoplasts. Thus, eqFP611 can be efficiently targeted to plant mitochondria, its subcellular localization being exclusively determined by the targeting information of the signal peptide fused to its N-terminus. Furthermore, this result confirms that eqFP611 is efficiently transported through two membranes while retaining its ability to fold properly for effective fluorescence. Similar to the native eqFP611, prolonged incubation of the protoplasts did not increase the intensity of the fluorescence.

The picture of the transfected protoplast displayed in Fig. 2A demonstrates nicely that the use of the MitoTracker filter set is appropriate to easily detect the red fluorescence of eqFP611 while effectively blocking chlorophyll autofluorescence. The latter is clearly visible through the FITC (fluorescein isothiocyanate) filter set (HQ 470/40/HQ 500 LP), which in turn blocks the fluorescence of eqFP611 (Fig. 2B). This autofluorescence in the chloroplasts exactly fits to the areas without fluorescence in Fig. 2A. Furthermore, the untransfected cells surrounding the eqFP611-expressing protoplast in Fig. 2A clearly show that no other autogenous fluorescence is visible through the MitoTracker filter set.

To assess the relative stability of the eqFP611 fluorescence in plants, we qualitatively compared the time elapsed until bleaching of the red fluorescence in protoplasts transiently expressing IVD145-eqFP611 and of MitoTracker^® ^Red CM-H2Xros (Molecular Probes, Eugene, OR) used for staining of untransfected protoplasts. This latter mitochondria-specific fluorescent dye has excitation/emission maxima of 579 nm and 599 nm, respectively. When individual cells of both approaches were inspected under identical light conditions in the fluorescence microscope, the fluorescence of IVD145-eqFP611 was at least as stable as the fluorescence of MitoTracker, which further demonstrates the usability of eqFP611 as marker at least in plant mitochondria.

### Co-expression of eqFP611 and smGFP4 in tobacco protoplasts

**Figure 3:**
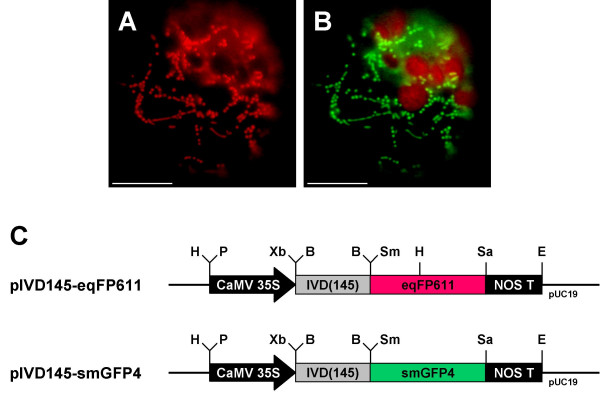
**Co-expression of eqFP611 and smGFP4 fusion proteins targeted to mitochondria**. Tobacco wild-type protoplasts transfected with plasmids pIVD145-eqFP611 and pIVD145-smGFP4. The eqFP611 and smGFP4 fusion proteins contain the mitochondrial presequence corresponding to the N-terminal 48 amino acids of IVD. Transfected protoplast seen through MitoTracker (A) and FITC (B) filter sets, respectively. Scale bars: 10 μm. (C) Map of plasmids pIVD145-eqFP611 and pIVD145-smGFP4. Black arrow, CaMV 35S: cauliflower mosaic virus 35S promoter; grey box, IVD(145): N-terminal 145 nucleotides of the IVD coding sequence; red box, eqFP611: eqFP611 coding sequence; green box, smGFP4: smGFP4 coding sequence; black box, NOS T: nopaline synthase terminator. H: HindIII, P: PstI, Xb: XbaI, B: BamHI, Sm: SmaI, Sa: SacI, E: EcoRI restriction sites. Vector backbone: pUC19.

Experiments like subcellular localization studies in which one of the fluorescent proteins is used to mark a distinct cellular compartment, require the simultaneous expression of two different fluorescent proteins. If eqFP611 is to be used routinely in such applications, its expression must be fully compatible with other IFPs, e.g. GFP. To test whether co-expression of both fluorescent proteins is indeed useful, tobacco protoplasts were simultaneously transfected with the constructs pIVD145-eqFP611 and pIVD145-smGFP4. Both plasmids contain identical mitochondrial targeting sequences fused to the N-termini of eqFP611 or smGFP4, respectively. Most of the succesfully transfected protoplasts incorporated both plasmids and expressed both eqFP611 and smGFP4. Identical patterns of the red and the green fluorescence in these protoplasts confirmed the co-expression of both proteins in the same cell (Fig. 3). In addition to the GFP-derived green fluorescence in the mitochondria, the red chlorophyll autofluorescence in the chloroplasts is seen with the FITC filter set (Fig. 3B).

**Figure 4:**
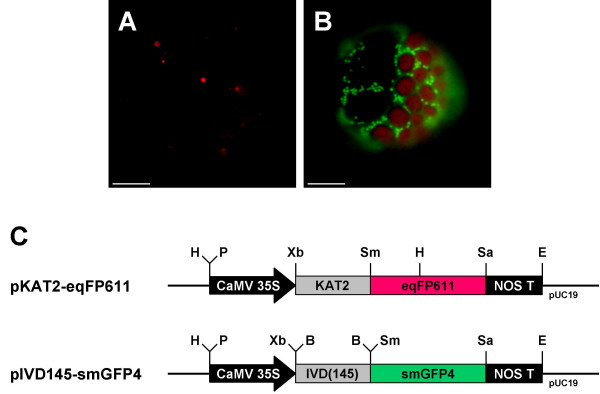
**Co-expression of peroxisomally targeted eqFP611 and mitochondrially targeted smGFP4**. Co-transfection of *N. tabacum *wild-type protoplasts with two separate plasmids encoding eqFP611 with a peroxisomal targeting signal 2 (pKAT2-eqFP611) and smGFP4 with a mitochondrial presequence (pIVD145-smGFP4). Images of a cell transfected with both constructs through MitoTracker (A) and FITC (B) filter sets, respectively. Scale bars: 10 μm. (C) Plasmids pKAT2-eqFP611 and pIVD145-eqFP611 used for transfection. Black arrow, CaMV 35S: cauliflower mosaic virus 35S promoter; grey box, KAT2: N-terminal 297 nucleotides of the KAT2 coding sequence; grey box, IVD(145): N-terminal 145 nucleotides of the IVD coding sequence; red box, eqFP611: eqFP611 coding sequence; green box, smGFP4: smGFP4 coding sequence; black box, NOS T: nopaline synthase terminator. H: HindIII, P: PstI, Xb: XbaI, Sm: SmaI, Sa: SacI, E: EcoRI, B: BamHI restriction sites. Vector backbone: pUC19.

To examine whether the transport into mitochondria of both fusion proteins occurs independently of each other and to exclude a possible chance "piggy back" effect during subcelluar transport of the two chimeric proteins, tobacco protoplasts were transfected with a different combination of plasmids. This time, pIVD145-smGFP4 was used for co-transfection with plasmid pKAT2-eqFP611, which latter encodes a recombinant protein of the peroxisomal targeting signal 2 (PTS2) [21] of 3-keto-acyl-CoA thiolase 2 (KAT2) [22] N-terminally fused to the eqFP611 reading frame. Red and green fluorescences were again found exclusively in the expected organelles (Fig. 4). The green fluorescence is observed in mitochondria, while the red fluorescence is visible in approximately 1 – 2 μm large roundish structures, a shape expected for leaf peroxisomes. No green fluorescence is seen in these organelles and conversely no red fluorescence is detected in mitochondria. This strongly suggests that if there is any interference, it does not disturb the correct targeting of the individual fusion proteins. Thus, eqFP611 and smGFP4 can be used in parallel to study protein sorting to different organelles within the same plant cell.

**Figure 5:**
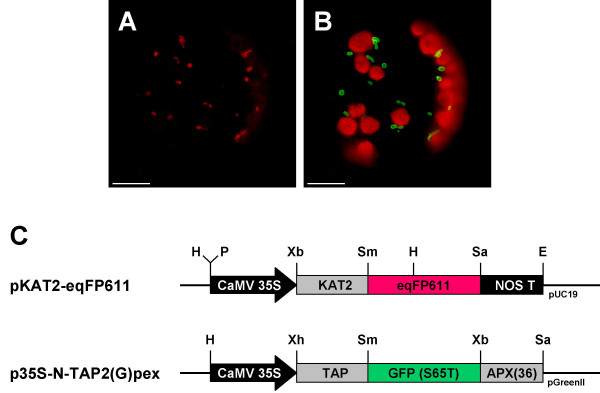
**Co-transfection of tobacco protoplasts with plasmids encoding eqFP611 and GFP targeted to peroxisomes**. Transfection of *N. tabacum *wild-type protoplasts with two separate plasmids encoding eqFP611 with a peroxisomal targeting signal 2 (pKAT2-eqFP611) and GFP targeted to the peroxisomal membrane (p35S-N-TAP2(G)pex). Pictures of the same protoplast taken through MitoTracker (A) and FITC (B) filter sets, respectively. Scale bars: 10 μM (C) Plasmid maps. Black arrow, CaMV 35S: cauliflower mosaic virus 35S promoter; grey box, KAT2: N-terminal 297 nucleotides of the KAT2 coding sequence; grey box, TAP: chimeric sequence for tandem affinity purification; red box, eqFP611: eqFP611 coding sequence; green box, GFP(S65T): GFP coding sequence including the S65T modification; grey box, APX: sequence encoding the C-terminal 36 amino acids of cotton ascorbate peroxidase; black box, NOS T: nopaline synthase terminator. H: HindIII, P: PstI, Xb: XbaI, Sm: SmaI, Sa: SacI, E: EcoRI, Xh: XhoI restrictions sites. Vector backbone: pUC19 and pGreenII, respectively.

To verify that the KAT2-eqFP611 fusion protein was indeed targeted to peroxisomes, pKAT2-eqFP611 was used for co-transfection together with p35S-N-TAP2(G)pex. The latter plasmid encodes a GFP fusion protein targeted to peroxisomal membranes by the C-terminal 36 amino acids of cotton ascorbate peroxidase (APX). As shown in Fig. 5, the patterns of the green and the red fluorescence overlap, indicating the correct peroxisomal localization of KAT2-eqFP611. Green fluorescence seems to be more intensive at the boundaries of the peroxisomes, while the red fluorescence is equally distributed within the organelles. This is consistent with the predicted intra-peroxisomal localization of the APX and KAT2 proteins, respectively. No green or red fluorescence is visible outside the peroxisomes. These experiments demonstrate that the N-terminal peroxisomal targeting signal 2 efficiently directs eqFP611 to the corresponding organelle and that this RFP can thus be exployed to study protein sorting into peroxisomes in plants.

Thus, as demonstrated by the expression in both mitochondria and peroxisomes, eqFP611 is a suitable partner for GFP in double-labeling experiments. When the two IFPs are co-expressed in the same cell, no mutual interference regarding development of fluorescence or intracellular sorting is observed. Additionally, both eqFP611 and GFP fluorescences can be easily distinguished by their emission spectra. The previously reported minor green fluorescence of eqFP611 was undetectable under the conditions used (Fig. 2B and 4B) [13].

Furthermore, despite the tendency of eqFP611 to form tetramers [13], its fusion proteins can be efficiently and reliably targeted to organelles. The transport across single (peroxisomes) or double (mitochondria) membranes does not interfere with the formation of the higher order structure necessary for emitting fluorescence. In addition, the fusion of a signal sequence to its N-terminus has no negative influence on the red fluorescence of eqFP611.

### Expression of both eqFP611 and smGFP4 from a single plasmid

**Figure 6:**
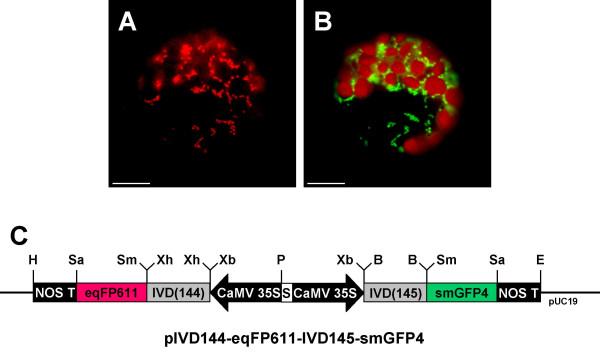
**Mitochondrially targeted eqFP611 and smGFP4 expressed from the same plasmid**. Transfection of *N. tabacum *wild-type protoplasts with a construct encoding both eqFP611 and smGFP4 with mitochondrial presequences (pIVD144-eqFP611-IVD145-smGFP4). Pictures of the same cell, taken through MitoTracker (A) or FITC (B) filter sets, respectively. Scale bars: 10 μm. (C) Plasmid used for transfection. Black arrow, CaMV 35S: cauliflower mosaic virus 35S promoter; grey boxes, IVD(144)/(145): N-terminal 145 and 144 nucleotides of the IVD coding sequence, respectively; red box, eqFP611: eqFP611 coding sequence; green box, smGFP4: smGFP4 coding sequence; black box, NOS T: nopaline synthase terminator; white box, S: spacer sequence. H: HindIII, Sa: SacI, Sm: SmaI, Xh: XhoI, Xb: XbaI, P: PstI, B: BamHI, E: EcoRI restriction sites. Vector backbone: pUC19.

Transformation of *Nicotiana benthamiana *leaves by injection of *Agrobacterium tumefaciens *[23] containing IFP fusion genes is another fast and simple method for the analysis of the subcellular localization of a protein. This procedure is presumably closer to the *in vivo *conditions than protoplast transfection, since the transformed cells remain in the original tissue context. In addition, this approach does not require the relatively laborious preparation of protoplasts. In this case, expression of the two fusion proteins from the same plasmid is advantageous, since a single transformation event is sufficient to ensure that every transformed cell contains both IFP genes. Apart from that, expressing both fluorescent proteins from the same plasmid under identical promoters should generate equal amounts of RFP and GFP within a cell. The entire procedure should be easier since only a single construct has to be handled. To investigate the feasibilty of this procedure, plasmid pIVD144-eqFP611-IVD145-smGFP4 containing both the eqFP611 and the smGFP4 genes with mitochondrial presequences each under control of a CaMV 35S promoter was constructed and first tested by transfection of tobacco protoplasts. Again, both red and green fluorescence could easily be detected in the same cell (Fig. 6). The fluorescence is found exclusively in mitochondria, the patterns of both red and green fluorescence being identical. This result is indistinguishable from the experiment with the same eqFP611 and smGFP4 expression cassettes encoded on two different plasmids (Fig. 3), but this time every transfected protoplast expressed both eqFP611 and smGFP4.

**Figure 7:**
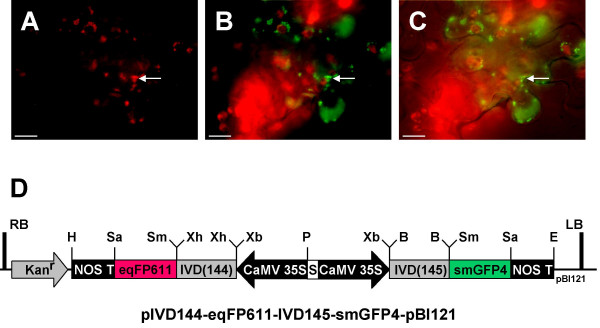
**Expression of eqFP611 and smGFP4 fusion proteins in *N. benthamiana *after leaf infiltration**. *Agrobacterium*-mediated transformation of *N. benthamiana *wild-type leaves with a construct encoding both eqFP611 and smGFP4 with mitochondrial presequences (pIVD144-eqFP611-IVD145-smGFP4-pBI121) on a single plasmid. Images of epidermal cell layers taken through MitoTracker (A) and FITC (B) filter sets, respectively. (C) Image taken through FITC filter set with addition of white light. Some mitochondria are examplarily indicated by a white arrow. Scale bars: 10 μm. (D) Representation of the plasmid used for agroinfiltration. The IFP expression cassettes are identical with those in Figure 6, but have been inserted into pBI121. Kan^r^: kanamycin resistance cassette (NOS promoter, neomycin phosphotransferase II, NOS terminator), RB: right border, LB: left border. Vector backbone: pBI121.

For co-expression of eqFP611 and smGFP4 in *N. benthamiana*, a binary vector suitable for plant transformation by agrobacteria was generated. The RFP-GFP-expression cassette from pIVD144-eqFP611-IVD145-smGFP4 was transferred into pBI121, creating pIVD144-eqFP611-IVD145-smGFP4-pBI121. *A. tumefaciens *containing the latter plasmid was then injected into *N. benthamiana *leaves. After transformation, both red and green fluorescence were visible in mitochondria of epidermal cell layers (Fig. 7), demonstrating the convenient use of the corresponding vector in this system.

### Tobacco plants stably expressing mitochondrially targeted eqFP611

**Figure 8:**
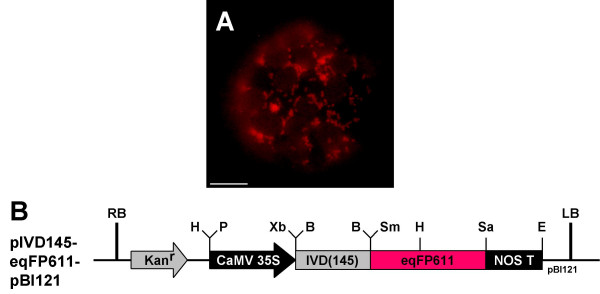
**Constitutive expression of mitochondrially targeted eqFP611**. Protoplasts derived from stably transformed *N. tabacum *plants constitutively expressing eqFP611 targeted to mitochondria (pIVD145-eqFP611-pBI121). (A) Image taken through MitoTracker filter set. Scale bar: 10 μm. (B) Plasmid used for transformation. The IFP expression cassette is identical with that in Figure 2, but has been inserted into pBI121. Kan^r^: kanamycin resistance cassette (NOS promoter, neomycin phosphotransferase II, NOS terminator). RB: right border, LB: left border. Vector backbone: pBI121.

A third way to use eqFP611 as a mitochondrial marker in plant cells is the generation of transgenic plants constitutively expressing mitochondrially targeted eqFP611. To create such plants, the RFP-expression cassette of pIVD145-eqFP611 was cloned into pBI121. The resulting plasmid pIVD145-eqFP611-pBI121 was stably transformed into tobacco by leaf disc transformation. Several independent plant lines were regenerated from transgenic calli and screened for bright red fluorescence in mitochondria. Red fluorescent mitochondria were observed in all T_0 _transformants, but expression levels varied between individual plants. In addition, segregation was observed in the next generation. Thus, only the offspring of the most strongly fluorescent T_1 _plant was used for propagation (Fig. 8). The transgenic plants completed their life cycle like wild-type plants and the red fluorescence in mitochondria was stably transmitted up to the T_3 _generation, the last generation analyzed. No phenotypic differences were observed between the transgenic and wild-type plants. Thus, eqFP611 obviously causes no cytotoxic or other detrimental effects even upon constitutive expression over several generations.

## Conclusion

Our results consistently demonstrate that eqFP611 meets all requirements for a potential fluorescent reporter protein for application in plants. It can be expressed in plant cells from the unmodified *E. quadricolor *cDNA sequence to levels easily detectable by epifluorescence microscopy without any adverse affect on viability. eqFP611 fluorescence can readily be separated from the red chlorophyll autofluorescence by using appropriate filter sets. Its subcellular localization can be efficiently controlled by N-terminal signal sequences. eqFP611 and GFP are fully compatible in dual-labeling experiments since there is no cross-interference with regard to expression and intra-cellular sorting and their fluorescence spectra can be clearly distinguished.

In addition, the plasmids created in the course of this work are convenient tools for the investigation of the subcellular localization of proteins in plant cells. The constructs encoding IFP fusions proteins with mitochondrial and peroxisomal targeting sequences can be used to express markers for the visualization of the corresponding organelles. The targeting sequences can also be easily exchanged to create new IFP fusions with any protein. Furthermore, all IFP expression cassettes can be transferred by HindIII/EcoRI digestion into the plant transformation vector pBI121 and derivatives thereof. Finally, the tobacco line stably expressing eqFP611 targeted to mitochondria is a useful source for protoplasts with an endogenous mitochondrial marker.

In summary, eqFP611 represents a true alternative to other RFPs and can be added into the tool box of red fluorescent proteins for use in plants.

## Methods

### Plasmid construction/cloning strategy

The eqFP611 wild-type coding sequence (696 bp) was PCR amplified from a respective cDNA clone [13] with primers eqFP611-H 5'-cacccgggatgaactcactgatcaagg-3' (in which the EcoRI site at nucleotide position 4 relative to the start codon was eliminated) and eqFP611-R 5'-tcgagctctcaaagacgtcccagtttg-3'. The PCR product was digested with XmaI and SacI and cloned into the respective site in the vector pIVD145-smGFP4 [17], in which eqFP611 replaced the smGFP4 gene. The resulting plasmid pIVD145-eqFP611 was used for studying mitochondrial targeting.

The plasmid peqFP611 for the expression of eqFP611 without presequence was obtained by excision of the IVD presequence from pIVD145-eqFP611 by BamHI digestion followed by religation.

To follow targeting into peroxisomes pKAT2-eqFP611 was constructed as follows: Primers KAT2-5'-2 5'-tctagaccatggagaaagcgatcgag-3' and KAT2-3'-2 5'-cccgggagggtcacctacttcacttgg-3' were used to amplify the N-terminal part (297 bp) of the 3-keto-acyl-CoA thiolase 2 (KAT2, At2g33150) coding sequence using total oligo(dT) primed cDNA from *A. thaliana *seedlings. The PCR product was cloned using the pGEM^®^-T Vector System I kit (Promega), sequenced, excised with XbaI and SmaI and ligated into plasmid peqFP611. The 99 amino-acid long N-terminal part from KAT2 including the peroxisomal targeting signal 2 (from amino acids 1 to 34) is now fused in frame upstream the eqFP611 coding sequence [21,22].

To study subcellular targeting of two fusion proteins simultaneously, a plasmid carrying two genes for different fluorescent proteins fused to identical mitochondrial targeting sequences (pIVD144-eqFP611-IVD145-smGFP4) was constructed. Briefly, IVD-eqFP611 and IVD-smGFP4 fusions both under control of a CaMV 35S promoter were introduced into the same plasmid in head-to-head orientation separated by a spacer sequence. Both presequences can be exchanged separately by XhoI (eqFP611) and BamHI (smGFP4) restriction digestion, respectively. Cloning details are available on request.

For constitutive expression of eqFP611 and GFP fusion proteins in plants, plasmids suitable for agrobacteria-mediated transformation were constructed. To generate pIVD145-eqFP611-pBI121, the HindIII-EcoRI fragment containing the eqFP611 expression cassette was removed from plasmid pIVD145-eqFP611 by cutting with EcoRI and partial digestion with HindIII. This DNA fragment was ligated into pBI121 digested with the same enzymes, which replaces the GUS cassette in this vector.

An analogous approach was used to generate pIVD144-eqFP611-IVD145-smGFP4-pBI121 from pIVD144-eqFP611-IVD145-smGFP4 and pBI121, except that the HindIII digestion was complete.

The vector backbone of psmGFP4 (sometimes also designated psmGFP) has been reported to be based on pUC118 and to contain the sequence ggatccaaggagatataaca**atg**agt around the smGFP4 start codon (bold) [GenBank: U70495] [24]. Our plasmid psmGFP4 and all its derivatives deviate from the published configuration in some aspects. Sequencing of pIVD145-smGFP4 shows the sequence downstream of the CaMV 35S promoter to be tctagaggatcct**atg**...(IVD)... ggatcccgcccggg**atg**...(smGFP4)... (start codons in bold). PCRs with one primer binding in the vector backbone and the other one in the CaMV 35S promoter or smGFP4 coding sequence in our psmGFP4 clearly show that the multiple cloning site is not orientated like in pUC118 and pUC18 but like in pUC119 and pUC19 (data not shown).

The absence of a 473 bp fragment in a digestion of the plasmid pIVD144-eqFP611 with RsaI (data not shown) rather indicates a pUC19-like instead of a pUC119-like configuration of the psmGFP4-derived vector-backbone.

### Polymerase chain reactions

All PCRs were performed with BD Advantage™ 2 Polymerase Mix (Becton Dickinson GmbH, Heidelberg, Germany), Phusion™ High-Fidelity DNA Polymerase (BioCat GmbH, Heidelberg, Germany) or self-produced Taq polymerase, respectively. Amplifications were done in 22 to 35 cycles under conditions recommended by the manufacturer (BD Advantage 2, Phusion). Reactions with self-produced Taq polymerase were done following standard protocols [25].

All PCR-derived DNA fragments were sequenced after cloning, except the RFP-expression cassette in pIVD144-eqFP611-IVD145-smGFP4. In this case, only the IVD144 mitochondrial presequence was analyzed by sequencing.

### Transformation procedures

PEG-mediated transient transfection of protoplasts was essentially carried out as described previously [26]. For transfection with single constructs, 60 μg DNA were used. In case of simultaneous transfection with two separate plasmids, 30 μg to 60 μg of each plasmid DNA were used.

Transgenic *Nicotiana tabacum *L., cv Petit Havana plants were generated essentially as described elsewhere [27]. Expression of IVD145-eqFP611 in the T_0_, T_1_, T_2 _and T_3 _plants was followed by fluorescence microscopic analysis of parts of the lower epidermis of leaves.

Agrobacteria-mediated transformation of *N. benthamiana *by leaf infiltration was performed as described before [23].

Strain GV2260 of *A. tumefaciens *was used for experiments requiring T-DNA transfer.

### Fluorescence microscopy

A Carl Zeiss Axioplan I microscope and the axiovision software (Carl Zeiss, Oberkochen, Germany) were used for visualization and documentation of eqFP611 and GFP fluorescence. The microscope was equipped with FITC (fluorescein isothiocyanate) (HQ 470/40/HQ 500 LP) and MitoTracker (HQ545/30/HQ 610/75) filter sets obtained from AHF (Tübingen, Germany) for GFP and eqFP611 analysis, respectively.

## Authors' contributions

JF designed and constructed the plasmids, carried out the microscopic analyses and drafted the manuscript.

SB conceived and supervised the project and worked over the draft version of the manuscript.

## References

[B1] Prasher DC, Eckenrode VK, Ward WW, Prendergast FG, Cormier MJ (1992). Primary structure of the Aequorea victoria green-fluorescent protein. Gene.

[B2] Chalfie M, Tu Y, Euskirchen G, Ward WW, Prasher DC (1994). Green fluorescent protein as a marker for gene expression. Science.

[B3] Yang TT, Cheng L, Kain SR (1996). Optimized codon usage and chromophore mutations provide enhanced sensitivity with the green fluorescent protein. Nucleic Acids Res.

[B4] Lippincott-Schwartz J, Patterson GH (2003). Development and use of fluorescent protein markers in living cells. Science.

[B5] Tsien RY (1998). The green fluorescent protein. Annu Rev Biochem.

[B6] Verkhusha VV, Lukyanov KA (2004). The molecular properties and applications of Anthozoa fluorescent proteins and chromoproteins. Nat Biotechnol.

[B7] Shaner NC, Campbell RE, Steinbach PA, Giepmans BN, Palmer AE, Tsien RY (2004). Improved monomeric red, orange and yellow fluorescent proteins derived from Discosoma sp. red fluorescent protein. Nat Biotechnol.

[B8] Jach G, Binot E, Frings S, Luxa K, Schell J (2001). Use of red fluorescent protein from Discosoma sp. (DsRed) as a reporter for plant gene expression. Plant J.

[B9] Dietrich C, Maiss E (2002). Red fluorescent protein DsRed from Discosoma sp. as a reporter protein in higher plants. Biotechniques.

[B10] Goodin MM, Dietzgen RG, Schichnes D, Ruzin S, Jackson AO (2002). pGD vectors: versatile tools for the expression of green and red fluorescent protein fusions in agroinfiltrated plant leaves. Plant J.

[B11] Mirabella R, Franken C, van der Krogt GN, Bisseling T, Geurts R (2004). Use of the fluorescent timer DsRed-E5 as reporter to monitor dynamics of gene activity in plants. Plant Physiol.

[B12] Nishizawa K, Kita Y, Kitayama M, Ishimoto M (2006). A red fluorescent protein, DsRed2, as a visual reporter for transient expression and stable transformation in soybean. Plant Cell Rep.

[B13] Wiedenmann J, Schenk A, Röcker C, Girod A, Spindler KD, Nienhaus GU (2002). A far-red fluorescent protein with fast maturation and reduced oligomerization tendency from Entacmaea quadricolor (Anthozoa, Actinaria). Proc Natl Acad Sci USA.

[B14] Wiedenmann J, Vallone B, Renzi F, Nienhaus K, Ivanchenko S, Rocker C, Nienhaus GU (2005). Red fluorescent protein eqFP611 and its genetically engineered dimeric variants. J Biomed Opt.

[B15] Haseloff J, Siemering KR, Prasher DC, Hodge S (1997). Removal of a cryptic intron and subcellular localization of green fluorescent protein are required to mark transgenic Arabidopsis plants brightly. Proc Natl Acad Sci USA.

[B16] Köhler RH, Zipfel WR, Webb WW, Hanson MR (1997). The green fluorescent protein as a marker to visualize plant mitochondria in vivo. Plant J.

[B17] Däschner K, Couée I, Binder S (2001). The mitochondrial isovaleryl-coenzyme a dehydrogenase of arabidopsis oxidizes intermediates of leucine and valine catabolism. Plant Physiol.

[B18] Kruft V, Eubel H, Jansch L, Werhahn W, Braun HP (2001). Proteomic approach to identify novel mitochondrial proteins in Arabidopsis. Plant Physiol.

[B19] Millar AH, Sweetlove LJ, Giege P, Leaver CJ (2001). Analysis of the Arabidopsis mitochondrial proteome. Plant Physiol.

[B20] Heazlewood JL, Tonti-Filippini JS, Gout AM, Day DA, Whelan J, Millar AH (2004). Experimental analysis of the Arabidopsis mitochondrial proteome highlights signaling and regulatory components, provides assessment of targeting prediction programs, and indicates plant-specific mitochondrial proteins. Plant Cell.

[B21] Kato A, Hayashi M, Kondo M, Nishimura M (2000). Transport of peroxisomal proteins synthesized as large precursors in plants. Cell Biochem Biophys.

[B22] Germain V, Rylott EL, Larson TR, Sherson SM, Bechtold N, Carde JP, Bryce JH, Graham IA, Smith SM (2001). Requirement for 3-ketoacyl-CoA thiolase-2 in peroxisome development, fatty acid beta-oxidation and breakdown of triacylglycerol in lipid bodies of Arabidopsis seedlings. Plant J.

[B23] Voinnet O, Rivas S, Mestre P, Baulcombe D (2003). An enhanced transient expression system in plants based on suppression of gene silencing by the p19 protein of tomato bushy stunt virus. Plant J.

[B24] Davis SJ, Vierstra RD (1998). Soluble, highly fluorescent variants of green fluorescent protein (GFP) for use in higher plants. Plant Mol Biol.

[B25] Sambrook J, Russel DW (2001). Molecular Cloning: A Laboratory Manual.

[B26] Koop HU, Steinmüller K, Wagner H, Rossler C, Eibl C, Sacher L (1996). Integration of foreign sequences into the tobacco plastome via polyethylene glycol-mediated protoplast transformation. Planta.

[B27] Horsch R, Fry J, Hoffman N, Wallroth M, Eichholtz D, Rogers S, Fraley R (1985). A simple and general method for transferring genes into plants. Science.

